# Prevalence and associated factors of chronic undernutrition among under five children in Adama town, Central Ethiopia: a cross-sectional study design

**DOI:** 10.1186/s13104-019-4552-1

**Published:** 2019-08-20

**Authors:** Jalane Mekonen, Samrawit Addisu, Hussen Mekonnen

**Affiliations:** 1 Department of Nursing and Midwifery, College of Medicine and Health Sciences, Debre Berhan University, P.O. Box: 445, Debre Berhan, Ethiopia; 2 College of Allied Health Science, Addis Ababa University, Addis Ababa, Ethiopia

**Keywords:** Child undernutrition, Stunting, Factors, Adama

## Abstract

**Objectives:**

The purpose of this study was to assess the prevalence and factors associated with stunting among under five children in Adama town, Central Ethiopia, 2013. A community based cross sectional study was conducted on 616 parent child pairs of under five children using structured questionnaire and anthropometric measurements. World health organization new growth reference was used to convert height measurements into Z-scores of the height for age indices considering age and sex of the children. Bivariate and multivariate logistic regression analysis were performed at *P* value < 0.05.

**Results:**

This study revealed that 44.4% of under five children were stunted. The findings showed that a significant positive association between stunting and mother educational status (AOR = 3.69 95% CI 1.42, 9.58), number of under five children in the house hold (AOR = 2.8 95% CI 1.77, 4.42), decision making on the use of money only by husband (AOR = 4.43 95% CI 2.51, 7.80), age of complementary foods started (AOR = 7.52 95% CI 3.39, 16.68), presence of diarrhea in the last two week (AOR = 1.79 95% CI 1.13, 2.83). Therefore, this study recommends intervention strategies focusing on encouraging women education, family planning and education on child caring practice

## Introduction

Chronic malnutrition is one of the leading global health concern, it is usually measured by stunting. Stunting, a length or height-for-age z score below minus two, is used by world health organization (WHO) as growth reference standard [1]. It is indicating an image of the previous nutritional background, and the current environmental and socioeconomic conditions. Stunting has negative effect on the cognitive development of a children with decreasing general school performance [2, 3].

The global proportion of stunted children were reduced slowly since 1990, however, in 2013, about 161 million children under 5 years were stunted [4]. In sub-Saharan Africa nearly 42% of under five children were stunted [5]. According to the Ethiopian Demographic and Heath survey conducted cross different region in 2011, 44% of children under 5 years found to be stunted of which 21% were severely stunted. This survey also showed 41.4% under five children being stunted in Oromia region of which 18.1% were severely stunted [6].

Around 45% of under five children death in the world is due to various forms of malnutrition, from them stunting is a major contributor [7]. Child malnutrition is one of the most serious public health problems in the developing countries such as Ethiopia. About 57% of child mortality in Ethiopia is due to malnutrition with some of the highest rates of stunting and underweight [8].

Stunting occurs due to different dimensional etiology with no single root cause, therefore, the intervention requires variety of ideas and collaboration of different sectors [9]. Past studies conducted in Ethiopia reported several factors associated to stunting. These includes age and sex of child, parents education and socio economic status, breastfeeding duration, complimentary foods, diarrhea episode, access to health care [10–12]. Therefore, this study aimed to assess the prevalence and factors associated with stunting among under five children in Adama town, Ethiopia. The study identifies significant risk factors of stunting which can support in targeting public health efforts to address the problem.

## Main text

### Methods

#### Study design, setting and participants

A community based cross-sectional study was conducted on 616 parent child pairs of under five children in Adama town, Ethiopia from March to April 2013. Adama town is located 99 km south east of the capital city, Addis Ababa. Administratively, Adama town is divided into 14 kebeles. With an estimated 155,349 population with 79,013 (50.8%) males and 76,336 (49.2%) females. Total numbers of under five children were 20,013 with 51.7% boys and 48.3% girls [13]. Under five children in randomly selected kebeles during the study period were included. Critically ill children and parents who were mentally incapable to provide consent information were excluded from the study.

#### Sample size and sampling procedures

The sample size was determined using a formula for estimation of single population proportion with the assumption of 95% confidence interval, 5% margin of error, and prevalence of chronic undernutrition in Oromia region at 41.4% [6], and design effect of 1.5. To compensate for the non-response rate, 10% of the determined sample was added up on the calculated sample size and the final sample size was found to be 616.

Multi stage sampling technique was used to get the required study subjects. First, four kebeles were selected from fourteen kebeles of Adama town using simple random sampling techniques. Second, the total households with under five children were also identified for each selected kebele. Third, the sample size was allocated for study areas of each selected kebele using population proportion technique. Finally, the households (HH) with under five children were identified using simple random sampling techniques. In the presence of more than one child one of them was selected by lottery method.

#### Data collection techniques and procedures

Interviewer administered questionnaires were employed to collect the data. The questionnaires were adapted and modified after reviewing relevant literature’s [8–12]. The English language questionnaire were translated into the regional language of Afan Oromo, and then translated back to English by bilingual persons to maintain the consistency of the questionnaires. Eight diploma level nurses and two BSc nurses degree were recruited for supervision of data collection after 3 days training. The questionnaires mainly focused on demographic, socioeconomic, dietary and health related factors.

Length was measured for children less than 2 years old and height was measured for those 2 years and older. Length was measured in supine position and height was measured in standing position with calibrated wooden length/height measuring board with sliding head bar. All the measurements were made in duplicate and recorded to the nearest 0.1 cm [14]. The height-for-age measurement status is expressed in standard deviation units which shows the deviation from the median of the reference population as recommended by WHO. Children with a measurement of < − 2 SD units from the median of the reference population were considered short for their age (stunted) and children with the measurement of < − 3 SD units from the median of the reference population were considered to be severely stunted [1]. Age of each child was collected from the parents and/or using vaccination cards.

#### Data processing and analysis

The children’s height was converted to the Z-score of height-for-age based on WHO growth reference population. Data was processed using EPi-info version 3.5.4 & WHO Antro version 3.2.2 to convert the nutritional data into Z-score of H/A taking age and sex into consideration then exported to SPSS version 20 for analysis. Descriptive statistics were used to determine the prevalence of stunting and frequencies and percentages were also calculated for all variables. Bivariate and multivariate logistic regression analysis methods were used to identify factors associated with stunting and to account for potential confounding factors.

### Results

#### Demographic and socioeconomic characteristics

A total of 610 under five children (304 girls and 306 boys) were enrolled in this study, with 99% response rate. Age wise, 19% of the study participants were in 6-11 months age group with the mean age of 23.56 months (± SD 17.54). More than half of the respondents (58.2%) follows Ethiopian Orthodox Christian in religion and 44.4% were from Oromo ethnic group. Majority of (84.4%) the respondents were married and 78.5% of the households were male headed. From the total number of mothers, 54.4% were house wife, 41% were completed elementary school. Regarding the fathers, 37.4% were private organization employee, while 30.7% were secondary school certificate holder. Two hundred thirty six (38.7%) of the respondents had a monthly income between 701 and 1000 ETH birr. Mean family size was 4.4 persons (± SD 1.61) while 17.5% of the households had more than five family size and about 42.6% of the households had more than two under 5 year children (Table 1).

**Table 1:** Socio-demographic characteristics of chronic undernutrition among under five children in Adama town, Central Ethiopia, 2013

Variables	Frequency	Percent
Child sex		
Male	306	50.2
Female	304	49.8
Head of the HH		
Male	479	78.5
Female	131	21.5
Father occupation		
Farmer	17	2.8
Merchant	71	11.6
Private	228	37.4
Government	123	20.2
Daily laborer	171	28.0
Mother occupation		
House wife	332	54.4
Farmer	5	0.8
Merchant	36	5.9
Private	74	12.1
Government	42	6.9
Daily laborer	121	19.8
Mother educational status		
Illiterate	84	13.8
Able read and write	46	7.5
Elementary	250	41.0
Secondary	167	27.4
Higher	63	10.3
Decision making on the use of money		
Mainly wife	106	17.4
Mainly husband	106	17.4
Only husband	218	35.7
Both jointly	180	29.5
Number of Family size		
2–5	503	82.5
6–12	105	17.2
> 12	2	0.3
Number of under five children		
1	350	57.4
2–3	260	42.6
Birth size		
Very large	46	7.5
Larger than average	76	12.5
Average	293	48.0
Smaller than average	103	16.9
Very small	92	15.1
Monthly income		
501–700 ETB	224	36.7
701–1000 ETB	236	38.7
1001–7000 ETB	150	24.6

#### Dietary and health related factors of chronic undernutrition

Almost all children (98%) were breastfed and 34.1% of children were exclusively breastfed for only four and less months. Mean duration of exclusive breastfed was 4.3 (± SD 1.97) months and the average duration of breastfed was 14 (± SD 8.83) months. About 29.5% breastfeed children received pre-lacteal foods/fluids and out of them 18.5% fed water. Majority of the children (81.5%) took additional foods/fluids in the past 48 h, out them 275 (45.1%) were started feeding within the age range of 4-6 months. Around 60.5% of children in the age group of 0–4 month were fed by using bottle. Almost all 598 (98%) respondents washed their hands whenever they fed the child, and also 40.3% washed their dishes immediately after use.

Majority (77.9%) of the children visited health facility for illness care; whereas 7.7% of the children did not receive any type of vaccine. Around 34.1% of the children had diarrhea in the last 2 weeks, out of them 32.2% having three to four episodes of diarrhea in a year. Three hundred twenty five (53.3%) of the children had fever before 2 weeks of the study period, likewise 39.8% of the children had ARI. Most of the house hold 254 (41.6%) throw the waste disposal in open field, followed by burning (20.6%) and common pit (19.2%) (Table 2).

**Table 2:** Dietary and health related factors of chronic undernutrition among under five children in Adama town, Central Ethiopia, 2013

Characteristics	Frequency	Percent
Breastfed ever		
Yes	598	98.0
No	12	2.0
Pre-lacteal foods/fluids^a^		
Yes	180	29.5
No	418	68.5
Colostrum^a^		
Yes	448	73.4
No	150	24.6
Type of pre-lacteal food/fluid^a^		
Water	113	18.5
Butter	7	1.1
Milk	58	9.5
Other	2	0.3
Still breast-feeding^a^		
Yes	328	53.8
No	270	44.3
Frequency of breastfeeding/day^a^		
< 8 times	104	17.0
8 times	80	13.3
> 8 times	144	23.6
Duration of exclusive breast-feeding^a^		
< 4 month	204	34.1
4–6 month	383	62.8
> 6 month	11	1.8
Duration of breast-feeding^a^		
< 12 month	347	56.9
12–24 month	205	34.2
> 24 month	46	7.5
Additional food/fluid in the past 48 h		
Yes	497	81.5
No	113	18.5
Age complementary food started^a^		
< 4 month	124	20.3
4–6 month	275	45.1
< 6 month	98	16.1
Wash hands whenever you feed the child		
Yes	598	98.0
No	12	2.0
Health facility for child sickness		
Yes	475	77.9
No	135	22.1
Immunization		
Yes	563	92.3
No	47	7.7
Diarrhea in the last 2 weeks		
Yes	208	34.1
No	402	65.9
Frequency of diarrhea/year^a^		
Once	16	7.7
Twice	106	51
3–4	67	32.2
5–18	19	9.1
Fever in the last 2 weeks		
Yes	325	53.3
No	285	46.7
ARI in the last 2 weeks		
Yes	243	39.8
No	367	60.2
Measles		
Yes	6	1.0
No	604	99.0

^a^n is not 610

#### Percentage distribution of chronic undernutrition

The percentage of children who were stunted (< − 2 SD) was 44.4%, of which 12.8% severely stunted (< − 3 SD). Out of the total children who are stunted, 147 (54.2%) were boys and 124 (20.7%) of the children was in the age group of 6–11 months.

#### Factors associated with chronic undernutrition

In bivariate analysis; the number of under five children, mother educational status, decision making on the use of money, duration of exclusive breastfeeding, pre-lacteal foods/fluids, additional foods in the past 48 h, age of complementary food started, presence of diarrhea and fever in the last 2 weeks had statistically significant association with stunting.

Multivariable logistic regression analysis showed that children from household’s decision making on the use of money by only father were more stunted (AOR = 4.43 95% CI 2.51–7.80) than those children found in the household of decision making by both jointly. Stunting was 3.69 times higher among children whose mother was illiterate (AOR = 3.69 95% CI 1.42–9.58) than children whose mother finished higher education. Similarly, the presence of more than two under five children in the household had 2.8 times higher risk for stunting (AOR = 2.8 95% CI 1.77–4.42). Likewise, significantly higher risk of stunting was observed among children who started complementary foods/fluids less than 4 months of age as compared to the other groups (AOR = 7.52 95% CI 3.39–16.68). Children who had diarrhea before 2 weeks of the study period were 1.79 times stunted than children without diarrhea (Table 3).

**Table 3:** Predictors of chronic undernutrition (stunting) among under five children in Adama town, Central Ethiopia, 2013

Variables	Stunted	
	COR (95% CI)	AOR (95% CI)
Number of < 5 children		
1	1	1
2–3	2.8 (2.01, 3.91)	2.8 (1.77, 4.42)*
Mother educational status		
Illiterate	2.48 (1.26, 4.85)	3.69 (1.42, 9.58)*
Able read and write	0.17 (0.06, 0.47)	0.30 (0.09,1.00)
Elementary	0.90 (0.52, 1.57)	1.40 (0.67, 2.93)
Secondary	0.84 (0.47, 1.51)	1.10 (0.50, 2.41)
Higher	1	1
Decision making on the use of money		
Mainly wife	1.35 (0.81, 2.26)	1.51(0.72, 3.18)
Mainly husband	1.93 (1.17, 3.20)	1.52 (0.78, 2.94)
Only husband	4.27 (2.79, 6.54)	4.43 (2.51, 7.80)*
Both jointly	1	1
Duration of exclusive breast feeding		
< 4 months	1	1
4–6 months	0.58 (0.41, 0.81)	0.36 (0.17, 1.03)
> 6 months	0.32 (0.08, 1.25)	0.27 (0.04, 1.71)
Pre-lacteal foods/fluids		
Yes	1.42 (1.00, 2.01)	0.82 (0.48, 1.38)
No	1	1
Additional foods in the past 48 h		
Yes	2.16 (1.45, 3.20)	1.68 (0.67, 3.97)
No	1	1
Age complementary food started		
< 4 month	3.48(2.00, 6.07)	7.52 (3.39,16.68)*
4–6 month	1.45(0.90, 2.34)	2.38 (1.28, 4.42)*
> 6 month	1	1
Diarrhea in the last 2 weeks		
Yes	1.78(1.27, 2.50)	1.79 (1.13, 2.83)*
No	1	1
Fever in the last 2 weeks		
Yes	1.70 (1.06, 2.72)	1.31 (0.72, 2.41)
No	1	1

* Statistically significant (*P* value < 0.05); 1 = Reference category

### Discussion

This study illustrated that the prevalence of stunting was 44.4%, which was in line with the national figure of EDHS 2011 (44%) [6] and with the result reported in Vietnam (44.3%) [15]. However, the percentage was lower than study reported in Northern Ethiopia (46.9%) [10], Pakistan (61%) [16] and India-Bihar State (54%) [17]. Besides, the result was higher than Western Ethiopia (32.4%) [18], North West Ethiopia (43.2%) [11], Oromia Region figure of EDHS 2011 (41%) [6], Afghanistan (39.9%) [19] and Indonesia (38.4%) [12]. Several reasons might contribute for the slight variation among this studies including study period, age difference of the participants, and socio-demographic characteristics of the participants, inclusion of both urban and rural population and taking average for national report.

Considering education of mothers, the present study revealed that stunting was 3.7 times higher among children whose mother was illiterate (AOR = 3.69 95% CI 1.42–9.58) than children whose mother finished higher education. The result was consistent with studies conducted in Vietnam, India and Afghanistan [15, 17, 19]. This is because educated mother have more knowledge in child caring practice, optimal child feeding and health seeking behavior. The study also showed that the presence more than one under five children in the household, decision making on the use of money only by husband and presence of diarrhea before 2 weeks of the survey had significant association with stunting. This finding was in line with studies done in North West Ethiopia, Vietnam and Afghanistan [11, 15, 19].

Higher risk of stunting was observed among children who started complementary foods/fluids less than 4 months of age as compared to the other groups (AOR = 7.52 95% CI 3.39–16.68). On contrary, study done in west Gojam showed that stunting was higher among children who started complementary foods/fluids at the age of greater than 12 months [11]. This difference may indicate both early and late introduction of complementary foods/fluids had significant association with stunting. Bottle feeding in 60.5% of the children aged less than 4 months in this study might also be a contributing factor.

### Conclusion

This study demonstrated that the socio-demographic factors of the child were major determinants for chronic undernutrition. Therefore, this study recommends intervention focusing on encouraging women education, family planning and education on child caring practice may ultimately reduce the prevalence of stunting.

## Limitation of the study

- Dietary intake data is not included in this study since it needs follow up records.
- This study only focused on the two main factors of chronic undernutrition (family and child level factors).

## Data Availability

The data sets used during the current study is available from the corresponding author on reasonable request.

## Abbreviations

HFZ: height for age Z score
MOH: Ministry of Health
SD: standard deviation
SPSS: Statistical Package for Social Science
WHO: World Health Organization
EDHS: Ethiopia Demographic and Health Survey
